# The Many Faces of Elongator in Neurodevelopment and Disease

**DOI:** 10.3389/fnmol.2016.00115

**Published:** 2016-11-01

**Authors:** Marija Kojic, Brandon Wainwright

**Affiliations:** Genomics of Development and Disease Division, Institute for Molecular Bioscience, The University of QueenslandBrisbane, QLD, Australia

**Keywords:** Elongator complex, neurodevelopment, neurological disorders, tRNA modifications, translation

## Abstract

Development of the nervous system requires a variety of cellular activities, such as proliferation, migration, axonal outgrowth and guidance and synapse formation during the differentiation of neural precursors into mature neurons. Malfunction of these highly regulated and coordinated events results in various neurological diseases. The Elongator complex is a multi-subunit complex highly conserved in eukaryotes whose function has been implicated in the majority of cellular activities underlying neurodevelopment. These activities include cell motility, actin cytoskeleton organization, exocytosis, polarized secretion, intracellular trafficking and the maintenance of neural function. Several studies have associated mutations in Elongator subunits with the neurological disorders familial dysautonomia (FD), intellectual disability (ID), amyotrophic lateral sclerosis (ALS) and rolandic epilepsy (RE). Here, we review the various cellular activities assigned to this complex and discuss the implications for neural development and disease. Further research in this area has the potential to generate new diagnostic tools, better prevention strategies and more effective treatment options for a wide variety of neurological disorders.

## Introduction

There are over 600 known neurological disorders and we are still far from fully understanding and finding treatment options for the majority of them. Many neurological disorders are inherited diseases, arising from defects in the development of the nervous system. Neurodevelopment is a complex dynamic process regulated by various genetic and environmental factors. The process is comprised of a series of intricate and coordinated events, required for converting neural precursor cells into functional neurons. Deregulation of these complex processes leads to abnormal neurodevelopment and profound neurological dysfunction. Genetic analysis of various neurodevelopmental disorders, including familial dysautonomia (FD; Anderson et al., 2001; Slaugenhaupt et al., 2001; Cuajungco et al., 2003), intellectual disability (ID; Najmabadi et al., 2011; Cohen et al., 2015), amyotrophic lateral sclerosis (ALS; Simpson et al., 2009) and rolandic epilepsy (RE; Strug et al., 2009), have identified mutations in Elongator complex subunits, suggesting that this highly evolutionarily conserved complex plays an important role in regulating neurodevelopment. In eukaryotes, Elongator is associated with diverse cellular activities including transcriptional elongation (Hawkes et al., 2002; Kim et al., 2002), cytoplasmic kinase signaling (Holmberg et al., 2002; Close et al., 2006), exocytosis (Rahl et al., 2005), cytoskeletal organization (Johansen et al., 2008), tubulin acetylation (Creppe et al., 2009) and translation (Huang et al., 2005; Esberg et al., 2006; Johansson et al., 2008; Bauer et al., 2012). In this review, we discuss the growing experimental evidence supporting the importance of Elongator in cellular processes known to be crucially important for neurodevelopment and nervous system function.

## The Elongator Complex

**Figure 1:**
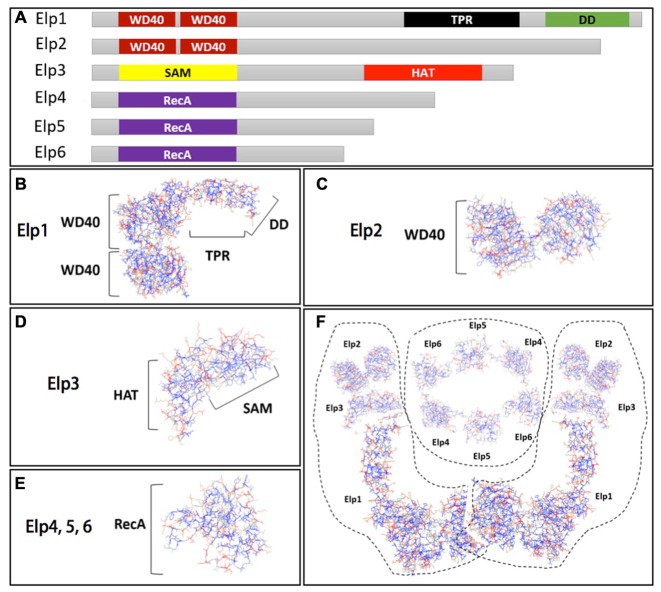
**The Elongator complex architecture. (A)** Schematic view of Elongator subunits (Elp1–6) and their domain structure highlighted by different colors. Structural model of: **(B)** Elp1 harboring two WD40 propeller domains, tetratricopeptide repeat (TRP) and DD domain; **(C)** Elp2 containing two WD40 propeller domains; **(D)** Elp3 with histone acetyl-transferase (HAT) and S-adenosyl-L-methionine (SAM) domain **(E)** and Elp4, 5 and 6 subunits that share a RecA fold. **(F)** The Elongator complex assembly in dodecamer with two Elp123 trimeric sub-complexes peripherally attached to the ring-like hexameric Elp456 sub-complex. Structural models of Elp1–6 were prepared using program Phyre2 (Kelley and Sternberg, 2009).

The Elongator complex consists of six subunits (Elp1–Elp6), which are organized into two three-subunit sub-complexes: the core sub-complex Elp123 (Elp1–Elp3), and the accessory sub-complex Elp456 (Elp4–Elp6; Otero et al., 1999; Li et al., 2001; Winkler et al., 2001). Each Elongator subunit is structurally well characterized in yeast (Figure 1A). Elp1 is the largest of the six subunits and acts as a scaffold for other Elongator proteins. This subunit harbors several WD40 repeats within two WD40 propeller domains, and one tetratricopeptide repeat (TRP) domain that binds specific peptide ligands and mediates protein–protein interactions (Cortajarena and Regan, 2006). An additional Elp1 domain has been recently identified: the C-terminus-localized dimerization domain (Xu et al., 2015; Figure 1B). Elp2 is the second largest subunit of Elongator complex with two WD40 propeller domains (Figure 1C; Fellows et al., 2000). Together with Elp1, Elp2 contributes to the stability of Elp123 sub-complex and integrates signals from different factors that regulate Elongator activity. Elp3 functions as the enzymatic core of Elongator, harboring two domains essential for Elongator function. These include: the S-adenosyl-L-methionine (SAM) binding domain required to catalyze a variety of radical reactions (Paraskevopoulou et al., 2006), and the histone acetyl-transferase (HAT) domain (Figure 1D; Wittschieben et al., 1999). Elongator subunits Elp4–6 share a RecA-like fold (Figure 1E) and assemble into a heterohexameric, ring-like structure. Glatt et al. (2012) showed that Elp4, 5 and 6 specifically bind to the anti-codon loop of transfer RNAs (tRNAs) and preserve ATPase activity, likely as means to control tRNA binding and release. The interaction of Elongator subunits and complex assembly has been reported by two separate studies, both proposing that the Elp456 heterohexamer bridges two peripherally attached Elp123 sub-complexes. These data indicate that Elongator is a dodecameric complex containing two copies of each of the six Elongator subunits (Figure 1F; Glatt et al., 2012; Lin et al., 2012). Elongator subunits are evolutionarily highly conserved from yeast to humans both in their sequence and interaction with other subunits. Conserved function across all species has been clearly demonstrated using a variety of different cross-species rescue experiments (Li et al., 2005; Chen et al., 2006, 2009). Deletion of any of the genes encoding the six subunits confers almost identical biochemical phenotypes in yeast (Fellows et al., 2000; Winkler et al., 2001; Frohloff et al., 2003), suggesting that there is a tight functional association between the proteins comprising the Elongator complex, and that the functional integrity of Elongator is compromised in the absence of any of its subunits.

The Elongator complex has been reported to orchestrate multiple functions across diverse organisms. Several loss-of-function studies have illustrated a key role for this complex in development by regulating a variety of different cellular activities. For example, yeast Elp mutants are hypersensitive to high temperature and osmotic conditions, presenting with defects in exocytosis, telomeric gene silencing, DNA damage response and adaption to new growth medium (Wittschieben et al., 1999; Rahl et al., 2005; Li et al., 2009; Chen et al., 2011). In *Arabidopsis thaliana*, mutations in Elp subunits resulted in impaired root growth (Nelissen et al., 2005), whilst deletion of Elp3 in *Drosophila melanogaster* was shown to be lethal at the larval stage (Walker et al., 2011). Elongator-deficient *Caenorhabditis elegans* exhibit defects in neurodevelopment (Solinger et al., 2010). In mice, a transgenic Elp1 knockout resulted in embryonic lethality due to failed neurulation and vascular system formation (Chen et al., 2009). Furthermore, several human neurological disorders have been linked to a deficiency of the Elongator, which will be discussed in more detail below.

The substrate specificity of Elongator remains controversial, and the definite number of roles this complex plays in different cellular activities is still to be confirmed (also reviewed in Svejstrup, 2007; Versées et al., 2010; Glatt and Müller, 2013). The complex was initially identified in yeast as the major component of RNA polymerase II (RNAPII) holo-enzyme (Otero et al., 1999; Wittschieben et al., 1999). *In vitro* studies using the HeLa cell line further confirmed that Elongator directly interacts with RNAPII and facilitates transcription in a chromatin- and acetyl-CoA-dependent manner (Hawkes et al., 2002; Kim et al., 2002). The Elongator complex has also been reported to play two other distinct nuclear roles. Knockdown of Elp3 has been shown to impair paternal DNA demethylation in mouse zygotes, a process that requires the Elp3 SAM domain (Okada et al., 2010). The complex was also demonstrated to be involved in microRNA (miRNA) biogenesis in *Arabidopsis*, whereby Elongator is believed to play a role in coupling the transcription of primary miRNAs and their subsequent processing (Fang et al., 2015). The majority of Elongator has been found to be located in the cytoplasm, consistent with the various cellular processes assigned to this complex that take place in the cytosol. Two studies have reported that Elongator regulates cytoplasmic kinase signaling through its interaction with c-Jun N-terminal kinase (JNK; Holmberg et al., 2002; Close et al., 2006). Holmberg et al. (2002) showed that Elongator is involved in the assembly of JNK-MAPK module through the association of Elp1 with JNK, resulting in JNK activation. Rahl et al. (2005) proposed an active requirement for the Elongator complex in the establishment and maintenance of yeast cell polarity, and in exocytosis, through its interaction with Rab GTPase Sec2p. As suggested by the authors, Elongator negatively regulates Sec2p-dependent, polarized secretion through a transcription-independent pathway. Johansen et al. (2008) proposed a model in which Elp1-assisted localization of filamin A into membrane ruffles regulates neuron migration in rats. Another study linked Elongator to the process of cytoskeletal organization and cell motility by demonstrating acetylation of α-tubulin by this complex in murine cortical neurons (Creppe et al., 2009). Although the Elongator complex has been implicated in the various cellular processes described here thus far, there is accumulating evidence in the last decade to indicate that the main role of this complex is to maintain translational fidelity via regulation of tRNA modifications. In eukaryotes, U_34_ in the anticodons of tRNA^Lys^_UUU_, tRNA^Glu^_UUC_ and tRNA^Gln^_UUG_ are modified to 5-carbamoyl-methyl-uridine (ncm^5^U), 5-methoxy-carbonyl-methyl-uridine (mcm^5^U), or 5-methoxy-carbonyl-methyl-2-thio-uridine (mcm^5^s^2^U). A number of studies have reported that these modifications require the Elongator complex (Huang et al., 2005; Esberg et al., 2006; Johansson et al., 2008; Bauer et al., 2012). The methyl-group transfer to tRNA U_34_ by Elongator likely involves a SAM-mediated mechanism in conjunction with an electron transfer from a cofactor complex Kti11/Kti13 (Boal et al., 2011; Kolaj-Robin et al., 2015). However, the precise molecular mechanism that underlies the tRNA modification by this complex is yet to be elucidated.

It is still unclear whether Elongator has many distinct functions in a cell or it regulates one process that leads to different downstream effects, via altered translation. Interestingly, Esberg et al. (2006) found that elevated levels of two tRNA species bypass all the *in vivo* requirements of Elongator in transcription and exocytosis. All the phenotypes of Elongator-deficient yeast cells can be suppressed by overexpression of tRNA^Lys^_UUU_ and tRNA^Glu^_UUC_ (Esberg et al., 2006). A recent study by Bauer et al. (2012) demonstrated that translation of a large number of proteins is regulated by Elongator and that cell division is under translational control of this complex. The most recent finding in *Caenorhabditis elegans* shows that Elongator is not a direct tubulin acetytransferase, but it rather regulates the expression of α-tubulin acetyltransferase at translational level, through its elevated AAA codon content and tRNA modification (Bauer and Hermand, 2012). The other cell activities regulated by Elongator might also be explained by its tRNA modification role and codon-dependent regulation of translation, which future experiments will elucidate.

## Role of Elongator in Neurodevelopment

**Figure 2:**
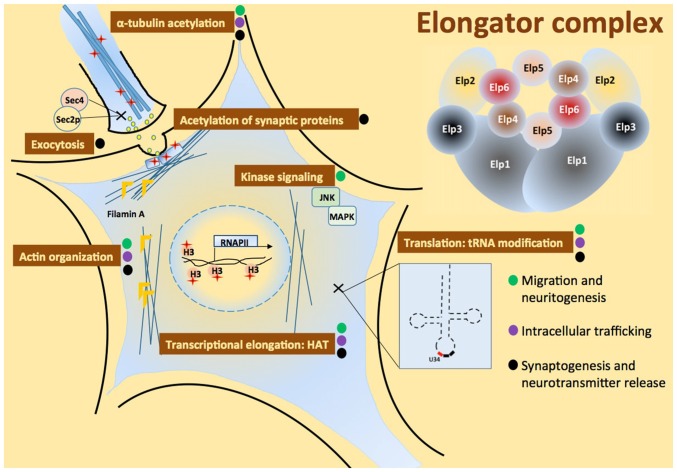
**Multiple roles of the Elongator complex in neurons.** The six-subunit Elongator complex is proposed to play multiple roles in cell: a role of histone-acetyl transferase (HAT) required for transcriptional elongation, it assists c-Jun N-terminal kinase (JNK)-MAPK module assembly, negatively regulates exocytosis through Sec2p-Sec4 interaction, regulates actin organization via Filamin A, promotes α-tubulin and synaptic proteins acetylation, and modifies transfer RNA (tRNA) U_34_. Elongator functions can be associated to cell activities critical for development and maintenance of the nervous system: migration and branching of neurons, intracellular trafficking and synapse formation, marked with green, purple and black circle in the figure, respectively.

Neural development is a complex process that requires neural induction, migration, differentiation, axon guidance and synapse formation. Both cell motility and the actin cytoskeleton play a central role in regulating how neuronal precursors proliferate and migrate to different parts of the developing brain. Once neural precursors have reached their final destination they undergo the process of differentiation, which involves the production and extension of axons and dendrites to form synapses, resulting in the establishment of functional neural circuits. Synapses are specialized sites of cell–cell contact where electrical signals trigger the release of neurotransmitters, which in turn activates postsynaptic receptors (Haucke et al., 2011). This highly regulated process is based on cytoskeletal organization, vesicular trafficking and polarized exocytosis. Here, we review the roles of Elongator during neurodevelopment, from transcription to translation (Figure 2).

Neurodevelopmental processes are dependent upon a broad number of factors regulating the expression of hundreds of genes controlling the terminal differentiation of neurons. The majority of genes activated during developmental processes are regulated at the level of transcriptional elongation (Muse et al., 2007). Elongator has been linked to the transcriptional regulation of several genes critical to various neurodevelopmental processes. Studies in human 293T (Han et al., 2007) and HeLa (Li et al., 2011) cell lines clearly demonstrate a role for the Elongator complex in the transcriptional elongation of heat shock protein 70 (HSP70), a gene that plays an important role in protecting of cells from apoptotic stimuli and in the stabilization of protein structures (Jäättelä et al., 1992; Mosser et al., 1997; Han et al., 2007). HSPs are developmentally regulated in the nervous system. HSP70 has been suggested to play an important role in both developing and adult mammalian brain and its expression within the nervous system is significantly higher compared to non-neuronal tissue (Manzerra et al., 1997). HSP70 has a neuroprotective role (attenuation of toxicity in a variety of neurodegenerative disease models (Arawaka et al., 2010)), and plays a role in axonal transport and neuronal signaling (de Waegh and Brady, 1989; Houenou et al., 1996; Thekkuveettil and Lakhotia, 1996). Elongator is also required for the activation of several genes involved in cell migration, such as those coding for the integrin receptor CD61, tenascin-C, and actin cytoskeleton modulators (Close et al., 2006). Integrins and tenascin-C are widely expressed in neuronal extracellular matrix during CNS development and they are shown to enhance neuronal precursors proliferation, migration and differentiation (Garcion et al., 2001; Flanagan et al., 2006). Taken together, these data show that the Elongator complex is responsible for the transcriptional regulation of a number of proteins that each plays a crucial role in various steps of development and maintenance of the nervous system.

The Elongator complex regulates a broad number of neurodevelopmental transcription-independent processes. Elongator activates JNK (Holmberg et al., 2002; Close et al., 2006), a stress-activated protein kinase that modulates the activity of a vast number of pathways. JNK signaling has been reported to be crucially important for neurodevelopment. JNK knockout studies in mice revealed its important role in brain morphogenesis, axonal specification and axon growth and guidance. In addition, JNK has been shown to govern synapse and memory formation (reviewed in Coffey, 2014).

Elongator is linked to synaptogenesis based on its role in vesicular trafficking and exocytosis via interacting with Rab proteins (Rahl et al., 2005). Rab proteins regulate membrane trafficking, which include vesicle formation, vesicle movement, and membrane fusion (Pfeffer, 2001). The yeast Rab protein Sec4p regulates exocytosis of post-Golgi secretory vesicles (Salminen and Novick, 1987). Sec2p is an essential protein that is recruited to sites of exocytosis, it targets the Sec4p activation event and facilitates polarized exocytosis (Walch-Solimena et al., 1997). Rahl et al. (2005) propose that the Elongator complex function in a cytosolic signal transduction pathway to regulate the localization of Sec2p and thereby the Rab activation event critical for polarized secretion. Rab proteins play a central role in neurodevelopment, by regulating the polarized neurite growth, axonal trafficking, and formation and maintenance of synapses (specific functions in synaptic vesicle exocytosis, reviewed in Ng and Tang, 2008).

The Elongator complex has also been shown to regulate migration of neural precursors through its interaction with filamin A, whereby Elongator is involved in the recruitment of filamin A in the membrane ruffles upon cell migration (Johansen et al., 2008). Elp3 was shown to localize to actin-rich domains at the edges of spreading HeLa cells (Barton et al., 2009). Filamin A organizes cortical actin filaments and dynamic three-dimensional networks in the leading edges of migrating cells and is essential for regulating the polarity of neocortical neurons during radial migration through the subventricular zone (SVZ) and intermediate zone (IZ) of the cerebrum (Nagano et al., 2004). Loss-of-function mutations in filamin A give rise to human periventricular heterotopia, a neurodevelopmental disorder caused by a failure of neurons to migrate to the cortex (Fox et al., 1998).

Acetylation of α-tubulin by the Elongator complex is yet another Elongator function that may play role in neural migration and branching (Creppe et al., 2009). In neurons the majority of cellular α-tubulin is acetylated. Creppe et al. (2009) demonstrated that lowering α-tubulin acetylation levels in microtubules through expression of α-tubulin K40A (a dominant-negative α-tubulin form that cannot be acetylated) recapitulated the migratory defects induced by Elp1/Elp3 silencing. The reduced acetylated α-tubulin levels seen upon Elongator deficiency in this study, suggest that this complex does not exclusively regulate cell motility via its association with filamin A, as migratory defects did not affect cell transition through neocortical SVZ and IZ or promote the formation of periventricular nodular heterotopia, nor via transcriptional elongation of key genes coding for proteins involved in cell migration, as the identity of these genes was cell-specific. The relationship between α-tubulin acetylation, neuron migration and branching is not yet clear but could rely on intracellular trafficking as α-tubulin acetylation is known to increase binding of motor proteins that regulate bidirectional molecular transport in axons and dendrites (Reed et al., 2006). The growing experimental and clinical evidence suggest that defective intracellular transport of specific proteins or organelles might be the hallmarks of several neurodegenerative processes, such as ALS, Parkinson’s disease, Huntington disease and Alzheimer’s disease (reviewed in Nguyen et al., 2010).

Elongator regulates neurotransmitter release and synapse formation, as demonstrated by one study showing that in *Drosophila* neurons, this complex is necessary and sufficient to acetylate Bruchpilot (BRP), an integral component of the presynaptic density where neurotransmitters are released (Miśkiewicz et al., 2011). BRP is a large cytoskeletal-like protein with its individual strands having their N termini facing the plasma membrane, contacting Ca^2+^ channels, and their C termini extending into cytoplasm capturing synaptic vesicles (Fouquet et al., 2009; Hallermann et al., 2010). Thus, BRP acts by concentrating synaptic vesicles at active zones, and facilitating synaptic transmission by establishing proximity between Ca^2+^ channels and vesicles to allow efficient transmitter release (Kittel et al., 2006; Hallermann et al., 2010). Miśkiewicz et al. (2011) suggest a model where Elongator is a BRP acetyltransferase and acetylation of BRP reorganizes its cytoplasmic tentacles, thereby regulating vesicle capturing by the C-terminal end of BRP and transport of vesicles at dense bodies. The local regulation of Elongator may enable single active zones to control neurotransmitter release and may hold the key to synaptic transmission regulation. The importance of protein acetylation in regulation of synapse composition and functionality in neurons has been demonstrated by recent study (Catarino et al., 2013). The possible role of Elongator in vesicular trafficking, exocytosis and synaptic transmission using above mentioned mechanisms remains to be tested in humans, yet evolutionary conservation of Elongator implies that it may play a similar role.

Recent studies have focused largely upon the pivotal role of Elongator in tRNA modification and these can be related to neurodevelopment in multiple ways. One recent report shows that Elongator is indeed linked to the translation of a variety of cell proteins, including those implicated in cell division (Bauer and Hermand, 2012). tRNA are more than simple adaptor molecules and have a surprising range of functions in the cell, such as tuning translation and protein expression in a tissue-specific manner and stress signaling, whereby these functions are all dependent on its modifications (Giegé, 2008; Thompson and Parker, 2009; Kirchner and Ignatova, 2015). Klassen et al. (2015) recently showed that the loss of wobble uridine modifications in the Elongator deficient yeast strain affects tRNA^Lys^_UUU_ function and results in a reduced total protein level. Moreover, the loss of these modifications in a subset of tRNAs was shown to lead to ribosome pausing at their cognate codons in both, yeast and* C. elegans* (Nedialkova and Leidel, 2015). Hence, upon Elongator malfunction, the kinetics of translation is perturbed, leading to the disruption of protein homeostasis in a cell and aggregate formation, as suggested by Nedialkova and associates. Neurons are particularly sensitive to the toxicity of misfolded proteins, hence, this could be the basis of neurodegenerative pathologies. In accordance with this, it can be postulated that Elongator-dependent tRNA modification regulate critical steps in biosynthesis and homeostasis of proteins required for the development and survival of specific neurons.

## Elongator Defects in Neurological Disorders

Mutations in Elongator complex subunits have been linked to different neuronal diseases (Table 1). *Elp1* mutations have been linked to an autosomal recessive disorder, FD (Anderson et al., 2001; Slaugenhaupt et al., 2001; Cuajungco et al., 2003). FD is among the most frequent hereditary sensory and autonomic neuropathies (Axelrod and Abularrage, 1982; Axelrod, 2002). The progressive degeneration of the sensory and autonomic nervous system in FD patients results in the following symptoms: cardiovascular dysfunction, pain insensitivity, gastrointestinal dysfunction, scoliosis, vomiting, defective lacrimation, extensive sweating and postural hypotension (Aguayo et al., 1971; Axelrod and Abularrage, 1982). Mortality in FD patients is high, and only 40% of patients survive beyond age 20 (Axelrod, 2004). FD patients have a mutation in the donor splice site of intron 20 of the *Elp1* gene, resulting in aberrant splicing (Anderson et al., 2001; Slaugenhaupt et al., 2001; Cuajungco et al., 2003). This mis-splicing results in tissue-specific exon skipping and consequently reduced levels of the Elp1 full-length protein. The FD mutation is incompletely penetrant, since the full length Elp1 is still synthesized albeit at lower levels in other cell types, whereas, in the central and peripheral nervous system only the truncated product is made (Slaugenhaupt et al., 2001; Cuajungco et al., 2003). This Elp1 truncated form cannot be detected in patients due to its degradation by the nonsense-mediated decay pathway (Cuajungco et al., 2003; Slaugenhaupt et al., 2004). Hence, Elp1 levels are very low in neuronal tissue from FD patients and unchanged in lymphoblasts for instance (Slaugenhaupt et al., 2001; Cuajungco et al., 2003). Heterozygous carriers also show reduced Elp1 expression but do not develop FD, suggesting the existence of an Elp1 tissue-specific threshold for the appropriate nervous system development and functioning. Experiments in HeLa, neuronal derived Elp1 RNAi cells and FD fibroblasts, showed that the Elp1-deficient cells exhibit defects in cell motility *in vitro* (Close et al., 2006). More recently, one group demonstrated that the cerebrum and fibroblasts from FD patients have reduced levels of mcm^5^s^2^ modifications at tRNA U_34_ (Karlsborn et al., 2014), which was further confirmed by Yoshida et al. (2015). This suggests that reduced levels of Elp1 due to aberrant splicing result in inefficient translation in FD, once again supporting the hypothesis that the predominant role of Elongator is the regulation of the rate of translation.

**Table 1:** **Elongator defects in neurological disorders**.

Neurological disorder	Affected Elp	Mutation	Reference
Familial dysautonomia	Elpl	Mutation in the donor splice site of intron 20	Anderson et al. (2001), Slaugenhaupt et al. (2001) and Cuajungco et al. (2003)
Intellectual disability	Elp2	Missense mutations	Najmabadi et al. (2011) and Cohen et al. (2015)
Amyotrophic lateral sclerosis	Elp3	Association with specific haplotype	Simpson et al. (2009)
Rolandic epilepsy	Elp4	Non-coding mutations	Strug et al. (2009)

ID is a disorder characterized by incomplete mental development, which results in limitations in both intellectual functioning and adaptive behavior (Schalock et al., 2010). A large-scale sequencing study identified missense mutations in the *Elp2* gene to be associated with ID or related neurological disabilities. Homozygous mutations in the *Elp2* gene were found in two families, each with three children suffering from moderate or severe IDs (Najmabadi et al., 2011). Cohen et al. (2015) recently reported on one more family with two brothers being affected by severe ID, spastic diplegia and self-injury. In both brothers, sequencing analysis found *Elp2* missense mutations to be linked with the inheritance of this disorder. One of the two *Elp2* gene mutations identified in this family was shown to have the same amino acid position as the recessive missense mutation in one of the two previously reported families (Najmabadi et al., 2011). The mechanism of this neurodevelopmental dysfunction may be related to the compromised function of the Elongator complex, due to the presence of a dysfunctional Elp2 subunit that normally acts as a signal-transducing platform. Thus, *Elp2* is likely to be a novel gene that has an important role in the development of recessive cognitive disorders, such as ID.

ALS, commonly called motor neuron disease, has been associated to allelic variants of *Elp3* gene (Simpson et al., 2009). ALS is a neurodegenerative disease characterized by progressive muscle weakness and atrophy due to degeneration of motor neurones in the primary motor cortex, corticospinal tracts, brainstem and spinal cord (Rowland and Shneider, 2001). Within 3 years of onset of the disease, respiratory muscle weakness generally results in death. The causative molecular pathway underlying ALS remains unknown and it is considered to be a complex disease caused by interplay between multiple mechanisms (Rowland and Shneider, 2001). Two independent studies performed by Simpson et al. (2009): a microsatellite-based genetic association study of ALS in humans and a mutagenesis screen in *Drosophila melanogaster*, identified allelic variants of *Elp3* gene as crucial for axonal biology. In the genetic association study, *Elp3* allelic variants were associated with ALS in three different populations. A mutagenesis screen in *Drosophila* identified *Elp3* mutations that conferred abnormal photoreceptor axonal targeting and neurodegeneration. In addition to this, Elp3 knockdown in zebrafish via morpholino technology resulted in dose-dependent shortening and abnormal branching of motor neurons. Therefore, understanding Elongator function is a promising route whereby we might gain insights into the mechanism of motor neuron degeneration in ALS.

*Elp4* mutations have been linked to RE, the most common human epilepsy, with onset at 7–10 years of age (Gomez and Klass, 1983). A neurodevelopmental disorder with epileptic focus typically located in the lower motor and/or somatosensory cortex (rolandic area; Koutroumanidis, 2007), RE is characterized by centro-temporal spikes (CTS) on an electroencephalogram. The common form of RE appears to have complex genetic inheritance, where the genetic contribution to the disease is yet to be elucidated. A recent study suggests that *Elp4* mutations are associated with CTS in RE families (Strug et al., 2009). A genome-wide association study in RE was conducted and fine mapping evidence pointed to the association of CTS with SNP markers in *Elp4* in both, discovery and replication data sets. Beside RE, there are other neurodevelopmental disorders associated with the CTS trait, such as speech disorder (Echenne et al., 1992) developmental coordination disorder (Scabar et al., 2006) and attention deficit-hyperactivity disorder (Holtmann et al., 2003) that could be also linked to *Elp4*, as in the case of RE. Strug et al. (2009) hypothesized that a non-coding mutation in *Elp4* gene impairs Elongator-mediated interaction of genes important for brain development, which leads to the susceptibility to seizures in neurodevelopmental disorders.

Given that Elongator has been shown to be involved in a variety of neurodevelopmental processes, this complex might be associated with a broad range of neurological disorders. Neurodegenerative diseases are mostly accompanied by transcriptional dysfunction, leading to neuronal death and in many of these cases, chromatin acetylation status is impaired by a mechanism related to the loss of HAT activity (Selvi et al., 2010). On the other hand, the Elongator association with neurological disorders could be through its tRNA modification role. Growing experimental evidence supports the link between tRNAs and neurological disorders (Kirchner and Ignatova, 2015). Several reports have associated ID and mutations in genes that encode for tRNA modification enzymes (Torres et al., 2014). A defect in Elongator complex-dependent tRNA modification could perturb translation in two different ways, by reducing the protein synthesis due to general slow-down of translation of lysine rich proteins that are found predominantly in the ribosomal machinery, or by leading to translational inaccuracy and protein misfolding. Neurons are known to be vulnerable to misfolded proteins, and the prion-like spread of pathogenic misfolded proteins probably holds a key as a general mechanism underlying neurodegeneration (Raj et al., 2012; Zhou et al., 2012). Warren et al. (2013) proposed the term “molecular nexopathy” to link accumulation of toxic protein aggregates with neural network disintegration, and argued that this can be a new paradigm of neurodegenerative diseases. Elongator-dependent regulation of translation could be an important factor contributing to this new paradigm.

## Concluding Remarks

The Elongator complex is a dodecamer composed of six subunits (Elp1–6) with two Elp123 trimeric sub-complexes peripherally attached to the ring-like hexameric Elp456 sub-complex (Glatt et al., 2012). It is a highly conserved complex among eukaryotes and its function is dependent on the integrity of all its subunits. Although a large number of cellular functions have been attributed to this complex, the predominant role of Elongator is in regulating tRNA modification. Yet a large amount of data exists to support a possibility that Elongator acts as a transcriptional regulator. Future high-resolution structural and functional studies aimed to identifying Elongator interactors, regulators, the molecular mechanism underlying its activity and substrate specificity will shed light on the true nature of this multi-subunit complex.

Elongator activity has been linked to a host of cellular processes crucial for neurodevelopment, including cytoskeletal organization, neuritogenesis, axon growth, axonal transport, neuronal signaling and cell motility. A role for Elongator in a number of distinct neurological disorders is emerging yet. The mechanism by which the perturbation of the complex leads to the specific neuropathogenic effects is yet to be defined. Once this is elucidated, exploration of methods to complement for Elongator dysfunction offers an approach to developing effective therapies for a variety of neurological disorders.

## Author Contributions

MK: main conception and design of the work, drafting the manuscript. BW: substantial contribution to the conception and design of the manuscript, critical revision of the work presented here. Both authors approve this manuscript to be published.

## Conflict of Interest Statement

The authors declare that the research was conducted in the absence of any commercial or financial relationships that could be construed as a potential conflict of interest.
